# Telemedicine in Heart Failure in the COVID-19 and Post-Pandemic Era: What Have We Learned?

**DOI:** 10.3390/biomedicines11082222

**Published:** 2023-08-08

**Authors:** Mateusz Sokolski, Marta Kalużna-Oleksy, Agnieszka Tycińska, Ewa A. Jankowska

**Affiliations:** 1 Institute of Heart Disease, Wrocław University Hospital, Wroclaw Medical University, 50-556 Wrocław, Poland; 2 Institute of Heart Diseases, University Hospital, 50-556 Wroclaw, Poland; 3 Department of Cardiology, Poznan University of Medical Sciences, 61-701 Poznań, Poland; 4 Department of Cardiology, Medical University of Bialystok, 15-089 Białystok, Poland

**Keywords:** heart failure, telemedicine, modern technologies, COVID-19, SARS-CoV-2, post COVID-19 era

## Abstract

Numerous studies showed that patients with heart failure (HF) and COVID-19 are at high risk of in-hospital complications and long-term mortality. Changes in the organisation of the medical system during the pandemic also worsened access to standard procedures, increasing the general mortality in HF and forcing the systems to be reorganised with the implementation and development of telemedical technologies. The main challenges for HF patients during the pandemic could be solved with new technologies aimed to limit the risk of SARS-CoV-2 transmission, optimise and titrate the therapy, prevent the progression and worsening of HF, and monitor patients with acute HF events in the course of and after COVID-19. Dedicated platforms, phone calls or video conferencing and consultation, and remote non-invasive and invasive cardiac monitoring became potential tools used to meet the aforementioned challenges. These solutions showed to be effective in the model of care for patients with HF and undoubtedly will be developed after the experience of the pandemic. However, the multitude of possibilities requires central coordination and collaboration between institutes with data protection and cost reimbursement to create effective mechanisms in HF management. It is crucial that lessons be learned from the pandemic experience to improve the quality of care for HF patients.

## 1. Introduction

Telemedical technology has developed rapidly in the last decade and is increasingly available in cardiology. Integrating telecare with routine clinical practice, through better efficiency, reduces costs and the risk of HF hospitalisation [1,2,3,4]. The severe acute respiratory syndrome coronavirus 2 (SARS-CoV-2), with around 700 million infections and 7 million deaths worldwide has caused extreme changes in the system of care in the recent years [5]. The coronavirus disease 2019 (COVID-19) strongly affected patients, especially those with cardiovascular diseases and heart failure (HF) in particular [6,7,8,9], and the use of telemedicine tools became advisable in this group [10,11,12]. HF patients should be assessed regularly with the evaluation of functional capacity, fluid status, cardiac rhythm, renal function, and review of medication every few months for patients whose condition is stable and more frequently for those who are unstable or have recently been decompensated [13]. Significant elements of optimal medical care are also the education processes and rehabilitation. Telemedicine minimises the risk of spreading SARS-CoV-2 and other seasonal viruses by avoiding travelling in person to care facilities yet continuing the therapeutic process. However, telephone assessments conducted according to the relatively scarce available clinical parameters can be insufficient. Remote monitoring (RM) can provide additional clinical data and help select high-risk patients. It has also become relevant for patients with suspicion of COVID-19 in the scenario when clinical presentation may overlap with COVID-19 infection or cause worsening HF [14,15]. Below is a list of the main aims in the management of HF patients in the COVID-19 era, which were supported by telemedicine:

- (L) limitation of SARS-CoV-2 and other pathogens transmission;
- (O) optimisation of the control visit;
- (U) up-titration of the therapy;
- (P) prevention of disease progression and HF worsening;
- (E) events of acute HF—monitoring of signs and symptoms in the course of COVID-19 and the post-COVID-19 period.

## 2. Social Media Platforms/Specifically Dedicated Platforms in the Era of the COVID-19 Pandemic

Social media (SoMe) platforms are among the most widely used sources of information in the world and are also one of the most effective ways to disseminate information. The importance of SoMe has changed to some extent in the COVID-19 era. SoMe became an important tool and resource of up-to-date scientific content with the possibility of discussing and opinion sharing between physicians including authors of the studies and authorities in the field. Direct connection via SoMe to the global cardiovascular community, the opportunity to discuss difficult cases and resolve clinical problems might improve the dissemination of knowledge.

The COVID-19 pandemic and the resulting global lockdown meant that many areas of life, including health care, had to completely change their functioning. Some visits were replaced with tele- or video visits, and meetings with professionals also moved to the virtual dimension. Both the management of patients and the exchange of experiences suddenly became difficult. It was reflected by a marked change in the search for information on the Internet. The peak of searches related to treatment options and medical resources was correlated with the peak of the incidence of COVID-19, whereas a negative correlation with the number of new cases was observed for searches concerning public health measures, social distancing, lockdown, and isolation. [16,17].

One of the disadvantages of social media is the difficulty in providing valuable information among the mass of misinformation or false content. Most of the content of SoMe is not peer-reviewed or verified. The amount of data on COVID-19 available on the Internet and SoMe has been so large that reliable data become practically unnoticeable. Such a situation is often called “Infodemia” [18,19]. The rapidly spreading information on SoMe requires responsible use if it is to be useful [20]. Moreover, SoMe is used to provide information and share content from cardiology journals, scientific meetings, or guidelines developed by scientific societies [21,22,23]. New studies can be independently discussed by authors and experts from all parts of the world on SoMe directly after the publication of results, anticipating the letters to the editor and confronting the results with other studies.

The roles of SoMe during the COVID-19 pandemic were as follows:

- Educational role—overcoming the lack of access to training and workshops;
- Enabling student-teacher contacts, particularly during the beginning of the pandemic when video-call platforms were not well-established yet;
- Amplifying public health messages—providing information to both patients and health professionals;
- Facilitating collaboration between professionals—discussion of test results, remote assessment of imaging tests, work of heart teams;
- Administrative role—staff recruitment, support, management;
- Providing social support—assistance in emotional relations, especially in realising that other people are in a similar situation [23,24].

## 3. Phone Calls/Live Video Conferencing/Electronic Consultations

Tele-visits have become particularly important in the time of the COVID-19 pandemic. They are useful both in patient care and in medical consultations between healthcare professionals (HCP). Telemedicine, in addition to the containment of viruses, prevents the deterioration of patient health due to misdiagnosis or inappropriate treatment of cardiovascular disease [25]. Medical advice and follow-up should be provided through telemedical solutions mainly in stable cardiovascular patients, and direct patient-doctor contact should only be limited to emergency situations. A telephone consultation makes it possible to monitor a patient’s condition and to improve compliance, especially in the case of patients the medical practitioner knows and with whom they have had recent contact in a medical facility setting. The possibility to issue an e-prescription or electronic sick leave cannot be overestimated. If possible (and necessary), a video consultation can be considered. The use of video seems to be particularly important in the management of patients with HF, where the objective assessment of the severity of HF signs by a physician is very important in the context of modifying treatment, including diuretics.

There are some telemedical systems which have been successfully deployed to evaluate and treat patients without referring them to in-person care [25], but none of them was created overnight for the pandemic situation. They were intended to utilise the potential of telemedicine in disasters and public health emergencies and did not contain a strategy for the management of cardiovascular patients in the specific circumstances of the pandemic era [26].

The use of telemedicine solutions is relatively simple and does not require the involvement of specialist equipment. Without exposing staff, tele-visits can be conducted with the use of commercial systems or paired tablets allowing communication with a clinician through a dedicated connection [25].

Telemedicine solutions are usually intended for patients with chronic diseases, but there are also solutions that can be used in hospital care as well as in acute or severe states. Electronic intensive care unit (ICU) monitoring systems allows to remotely observe the status of dozens patients in ICU in many hospitals and seems attractive and innovative tool for monitoring severe patients [25]. The clinical application of such technological and staffing complexities makes it impossible to create such a program on short notice, but rapid deployment of the two-tablet approach can reduce the number of healthcare workers.

Moreover, telemedicine can provide rapid access to subspecialists who are not immediately available in person to proceed with specialist consultation teams. Patients with HF usually have multiple comorbidities, hence multi-specialist care is important for them, and consultations of, for example, a pulmonologist or diabetologist with the use of telemedicine solutions allow the quick commencement or optimisation of accompanying treatment.

An exemplary solution is the pilot program E-Consultation, implemented in Poland in May 2023. The platform enables the consultation of patients treated by regional hospitals with tertiary centres. The platform stores test results in the cloud. All e-consultation decisions, such as hospital referrals, are electronically signed whereupon they become legally binding. This is a very beneficial change for patients who will not have to leave their place of residence to obtain a specialist’s opinion on their condition. The platform makes it possible to plan the diagnostic and therapeutic process and reduces the unnecessary transport of the patient. Despite great possibilities, telemedicine cannot replace in-person care provided by physicians and other professionals (nurses, medical assistants, physician assistants) but may improve communication, especially with other professionals. Teleconsultations may also reduce the cost of unjustified emergency department admissions.

When this pandemic is over, what should be taken into consideration are the technological factors impacting both patients and clinicians through the adoption of mobile health tools [27]. Patient involvement might be further needed to suggest usable and meaningful solutions.

## 4. Non-Invasive Monitoring

Telemedicine was a useful tool for managing patients with HF affected by SARS-CoV-2 in the prehospital phase, during hospitalisation, and after hospitalisation. The need for self-isolation, presentation to an emergency department, and urgent hospitalisation could all be supported with the existing virtual mediums. Finally, when patients were hospitalised in an acute condition, new technologies supported the medical team by minimising unnecessary contact or reducing the time of contact with an infected patient, in addition to providing information about their clinical status. Electronic stethoscopes and mobile ultrasound probes as part of point-of-care ultrasound devices for lung and pleural ultrasound and echocardiography also had an important place during in-hospital care. Assessment with lung sonography is a useful tool to visualise consolidations, atelectasis, interstitial oedema, pneumothorax, and pleural effusion. Problem-focused cardiac ultrasound performed at the bedside by the person providing care for the patient detects cardiac involvement and complications of COVID-19 [28,29]. The images can be recorded, stored, and subsequently analysed by an echocardiographer after wireless transfer to the hospital network. Video monitoring stations became standard in the in-hospital care of COVID-19 patients together with vital signs monitoring. Additionally, video conversations with patients and/or relatives were of great importance in the decision-making process, where patients and/or family members should play key roles, especially in COVID-19 circumstances [30]. The early post-discharge phase after an acute HF event is called the “vulnerable phase” due to the high number of adverse outcomes. Many patients after COVID-19 infection suffer from early complications too [31]. Therefore, close follow-up after hospitalisation is crucial to prevent readmission. Optimally, it can be combined with virtual monitoring and consultations. Several randomised controlled trials with non-invasive telemedical systems in HF have been performed and demonstrated inconsistent results [4,32,33,34]. It may be the case that clear benefits could not be achieved due to the limitations related to the nature of the measured parameters. The BMAD Trial: Wearable Remote Monitor Reduces Hospital Readmission Risk in HF Patients, presented at the American College of Cardiology’s Annual Scientific Session & Expo 2023, explored the role of thoracic fluid index measurement with a wearable non-invasive device. The use of the system improved quality of life, time to first HF hospitalisation, and time to the combined event of HF hospitalisation, emergency visit, or death (38% relative risk reduction, 7% absolute risk reduction at 90 days) [35]. The study was not randomised but has shown how weekly phone calls and monthly office visits together with non-invasive lung fluid measurement can be integrated during the COVID-19 pandemic era. There are also some suggestive differences in patient compliance and algorithms for therapeutic decisions based on the parameters obtained. The parameters usually obtained are symptoms (shortness of breath, oedema) and signs (heart rate, type of rhythm, blood pressure, weight, and pulse oximetry), which should be monitored in the course of and post-COVID-19 period. Finally, not every patient is able to obtain specific parameters due to limited access to the internet, which mainly refers to older patients with additional cognitive and sensory limitations.

## 5. Remote Monitoring of Cardiac Implantable Devices

Remote monitoring (RM) in patients with cardiac implantable electronic devices (CIEDs) has been possible for many years now. According to the 2021 ESC guidelines on cardiac pacing and cardiac resynchronization therapy, device-based RM is recommended in class I to reduce the number of in-office follow-ups in patients with pacemakers who have difficulties attending in-office visits and to enable early detection of events, particularly in patients who are at increased risk. Moreover, in patients on remote devices, in-office routine follow-up for single- and dual-chamber pacemakers may be spaced by up to 24 months in order to provide earlier detection of clinical problems or technical issues (class IIa of recommendations) [36]. The Expert Consensus Statement from 2015 suggests that all patients with CIED should be offered RM as part of a standard management strategy [37]. Based on the ESC 2021 HF guidelines, both non-invasive and invasive home monitoring may be considered (class IIb of recommendations) in order to improve clinical outcomes. Furthermore, the HF guidelines place the role of remote monitoring strategies in HF in the post-COVID-19 era among gaps in evidence [13]. All these documents, despite variable clinical trial results, together with rapid developments in technologies resulted in the availability of such a possibility for a significant number of patients in January 2020, at the moment of the pandemic outbreak. The medical reality changed significantly, and RM became necessary to effectively manage patients with HF in the COVID-19 pandemic era. ESC COVID-19 guidance suggests that remote interrogation or RM should be used as much as possible to replace routine visits to outpatient clinics [11]. This was especially relevant for patients who had not previously been under observation via remote interrogation/monitoring. First, it requires personnel and technical re-organisation for a telemetry-based model. Second, patients should be instructed and coached regarding the potentially unusual control system. Some devices require an in-office visit for RM activation and instruction on how to plug in the transmitter device. Patients with ventricular arrhythmia detection, implantable cardiac device (ICD) therapy, and lead dysfunction may require urgent in-office device interrogations and hospital admission. The control procedure should limit contact with other patients, and interrogation should preferably use wireless communication [38]. RM integrates information about arrhythmia burden, percentage of pacing, diurnal heart rate variability, patient activity, and lung fluid content (thoracic impedance, fluid index) which can help to identify patients at increased risk of subsequent HF hospitalisation [39]. Various parameters can be combined automatically by an algorithm calculating risk status and selecting high-risk patients, requiring close surveillance and frequent management [40,41]. The clinically available HeartLogic platform (Boston Scientific, Marlborough, MA, USA) with CIEDs algorithms which integrate thoracic impedance, respirations, heart rate, heart sounds and activity in order to detect early signs of acute HF was used to analyse the impact of COVID-19 on congestion and worsening HF [42]. The authors found a significant decrease in activity level and mean heart rate and only slight increases in thoracic impedance. The study generated the hypothesis that decreased autonomic tone, low patient activity and limited access to unhealthy food may have resulted in less congestion and acute HF events.

Another option can be implantable hemodynamic monitors. Pulmonary artery pressure monitoring was shown as an effective tool in reducing HF hospitalisations in patients with HF with reduced and preserved ejection fraction [43,44,45]. Although the number of implanted devices was low, there has been an increasing number of active cardioMEMS (Abbott Laboratories, Plymouth, MN, USA) patients from the beginning of the SARS-CoV-2 pandemic [46], and benefits from invasive monitoring were reported. Almufleh et al. showed the impact of COVID-19 on a cohort of patients with HF and implanted CardioMEMS system [47]. This effect was noted among HF patients with already implanted devices, during the first wave of the COVID-19 pandemic. The frequency of crossing pulmonary pressure thresholds increased with subsequent increase in clinician interventions and, finally, a reduction in hospitalisations due to HF. Similar results were reported for another retrospective study with wireless implantable hemodynamic monitoring (W-IHM) [48]. The cohort size and the retrospective nature of the study were the limitations of the study, and RM was probably only partly responsible for preventing hospitalisations. On the other hand, a recently published GUIDE-HF randomised control study showed a possible benefit of haemodynamic-guided management on the primary outcome in the pre-COVID-19 period, mainly consisting in a lower HF hospitalisation rate compared with the control group. However, this effect did not persist in the pandemic period [45]. The ambiguous results show the impact of the reorganisation of health care systems and the reduction of acute admission to the emergency and cardiology departments in general [49]. The above-mentioned effect can be multifactorial. Reduced daily activity, dietary limitations, isolation and fear may have a paradoxically positive effect on compliance, restriction on fluid intake and exposure to other infectious agents responsible for decreased risk of decompensation. The MONITOR-HF study checked the effect of haemodynamic monitoring of pulmonary artery pressure in the European healthcare system in patients treated with contemporary guideline-directed medical therapy. Haemodynamic monitoring improved quality of life measured with Kansas City Cardiomyopathy Questionnaire (KCCQ) score at 12 months and reduced HF hospitalisations and/or urgent visits requiring IV diuretics during follow-up (HR 0.56; 95% CI 0.38–0.84; *p* < 0.01) [50]. The limitation of the study was its open-label character and that it was performed only in one European country, The Netherlands, so it is still unknown how it would be implemented in real-life conditions across more European countries. Some insight has been brought by a multicentre analysis from Belgium, Switzerland, and Great Britain, using the same technology. The number of HF hospitalisations was reduced after 6 (34 vs. 17; *p* = 0.014) and 12 months (48 vs. 29; *p* = 0.032). HF-related healthcare costs were reduced from EUR 6286 to EUR 3761 at 6 months (*p* = 0.012) and from EUR 8960 to EUR 6167 at 12 months (*p* = 0.032) [51]. Apart from cost reduction, the main challenge for telemedicine remains the prevention and early detection of worsening HF events which is strongly recommended by the consensus statement of HFA [52].

## 6. Telerehabilitation

Cardiac rehabilitation (CR) reduces morbidity and mortality and improves the quality of life in cardiac patients [53,54,55]. Low awareness of this fact among patients and physicians and lack of space in CR centres, among other factors, may contribute to low referral and registration rates for CR [56]. The care programmes for cardiovascular patients should be comprehensive and include elements that will simplify contact with the patient and allow for continuity, e.g., after hospitalisations. Several barriers at the level of the patient, physiotherapist, and healthcare system that hinder the utilisation of CR and the sustainability of its effects may be solved through cardiac telerehabilitation (CTR) [56,57]. However, the selection of telemonitoring should be tailored via telemanagement individually for each patient, taking into account their clinical condition and comorbidities. The majority of guidelines of European and American scientific societies recommend the creation of a holistic model of treatment of patients with cardiovascular diseases including pharmacotherapy, treatment implantable devices with remote monitoring, cardiac rehabilitation, and long-term care, especially for patients with HF [13,58].

Many documents and position papers indicate a promising new direction of rehabilitation, i.e., hybrid cardiac telerehabilitation [59,60] or remotely monitored rehabilitation [61,62]. It is also worth mentioning the results of the randomised, multicentre, prospective TELEREH-HF (Hybrid comprehensive TELEREHabilitation in Heart Failure patients) study conducted in a group of 850 patients with HF, which evaluated comprehensive hybrid telerehabilitation (including telecare and remote monitoring implantable devices) [63]. It has been shown that a nine-week comprehensive hybrid telerehabilitation significantly improves physical efficiency and quality of life in patients with HF, in contrast to standard care [63]. It was neutral in terms of its impact on mortality and hospitalisation in long-term follow-up (14–26 months) with a reduction in total and cardiovascular mortality and a reduction in hospitalisation and was safe and well tolerated by patients [63,64]. Moreover, recent systematic reviews and meta-analyses show that multidisciplinary CTR or exercise-based CTR is a safe and at least equally (cost-) effective alternative to regular, centre-based CR in patients with coronary artery disese or chronic HF [65,66,67,68].

Telecardiology is part of an innovative model of outpatient care for patients with cardiovascular diseases, which may contribute to improving the prognosis and quality of life of those patients, as well as reduce the number of readmissions, leading to a beneficial effect both on patient health and on reducing the costs of the health care system. It is emphasised that telemonitoring as a form of routine management, including rehabilitation, may also reduce the spread of infection, including SARS-CoV-2 [69].

Furthermore, during the COVID-19 pandemic, the use of telecardiology in everyday practice has taken on a completely new dimension and has become the optimal solution, and sometimes the only form of implementation of diagnostic and therapeutic procedures, as well as prevention and rehabilitation [70].

## 7. Future Challenges and Limitations

**Figure 1:**
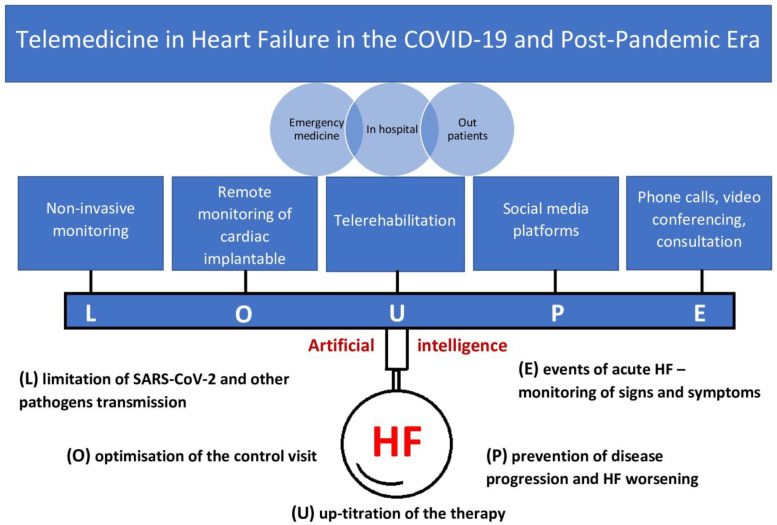
Study summary.

Key points of the current study are summarized as shown in Figure 1.

The latest observational studies and case reports generated hypotheses, which should be verified in larger cohort studies or randomised controlled clinical trials. More information is still needed concerning the effect of COVID-19 on HF presentation and HF management. The experience of the COVID-19 pandemic can result in a better understanding of HF patients and optimise clinical services now and during the recovery phase, as well as in the more distant future. There is a need to establish protocols and infrastructure to integrate new technologies with standard clinical care. Importantly, telecare cannot replace personal contact and standard clinical evaluation, and the overall strategy should be personalised by the HF team. The transfer of medicine to the virtual space may have psychosocial consequences for the patient. Direct interaction with a doctor provides a better insight into the clinical condition and builds trust in the patient–physician relationship. A patient deprived of this interaction can be frustrated, which may translate into the quality of care. Moreover, in some groups of patients, there is a risk of excluding individuals who are technology-averse or with limited access to the Internet for economic or technological reasons. Effective telemedicine requires real-time data access with uninterrupted and reliable connections. The healthcare system should offer equal accessibility for all patients regardless of socioeconomic status and age, involving medical caregivers and family and adapting the proposed technological solutions to the patient’s needs. The cost of the implementation of telemedical solutions can be a limitation for some healthcare systems. Therefore, it is necessary to base it on the cost-effectiveness analysis showing potential economic benefits. Telemedicine should not be limited to large centres and hospitals. The key is also the integration of small hospitals and ambulatory health centres and general practitioners. The information obtained via telemedicine should be appropriately interpreted and lead to adequate diagnostic and therapeutic actions, which seems to be the key element of successful virtual control. After the COVID-19 pandemic is over, many telemedicine solutions will most likely remain in the system of care for patients with cardiovascular diseases. The group of cardiological patients in which telemedicine is particularly important and allows for the optimisation of care are patients with HF. As a chronic disease, HF requires constant and frequent contact with medical personnel. Some of the visits and many forms of remote monitoring can find application in the model of care for patients with HF which will be developed after the experience of the pandemic period.

In the era of the development of new and drug-resistant pathogens, the experience of using telemedicine during the COVID pandemic is invaluable. It constitutes the basis for further intensive development of this area of medicine. In addition, telemedicine was found to be the future of the rapid development of mechanistic aspects of medicine, including artificial intelligence (AI)—an indispensable element of futurity. Telemedicine will generate a significant amount of data, requiring management, storage and interpretation. This concerns not only clinical data but also the results of laboratory and genetic tests, imaging examinations, devices (non-invasive, CIEDs, sensors), information obtained from individual patient measurements, medical records in systems, and social and environmental information. There is a need to find effective ways to handle “big data”, and AI brings a mechanism which can serve as a first line verification method, generating alerts and communication. A specific sub-discipline of AI, involved in diagnostic algorithm and outcome prediction, is machine learning algorithms (deep learning and neural networks) based on the trained processes generate information from data sets, being part of the decision-making process and identification of high risk states [71]. The implementation of various technologies and AI requires central legal regulations and standardisation to enable data exchange between different systems, but above all, AI should be integrated with human intelligence to reach the main aim which is improvement of the effectiveness and quality of care in HF [72].

## Data Availability

Not applicable.
